# Rewilding the Sea with Domesticated Seagrass

**DOI:** 10.1093/biosci/biab092

**Published:** 2021-09-22

**Authors:** Marieke M van Katwijk, Brigitta I van Tussenbroek, Steef V Hanssen, A Jan Hendriks, Lucien Hanssen

**Affiliations:** Department of Environmental Science, Institute for Water and Wetland Research, Radboud University, Nijmegen, The Netherlands; Reef Systems Unit, Instituto de Ciencias del Mar y Limnología, Universidad Nacional Autónoma de México, Puerto Morelos, Quintana Roo, Mexico; Deining Sustainable Coastal Zone Management, Nijmegen, The Netherlands; Department of Environmental Science, Institute for Water and Wetland Research, Radboud University, Nijmegen, The Netherlands; Department of Environmental Science, Institute for Water and Wetland Research, Radboud University, Nijmegen, The Netherlands

**Keywords:** ecosystem services, mariculture, native seed, reproductive biology, restoration, new domestication

## Abstract

It is well known that seagrass meadows sequester atmospheric carbon dioxide, protect coasts, provide nurseries for global fisheries, and enhance biodiversity. Large-scale restoration of lost seagrass meadows is urgently needed to revive these planetary ecosystem services, but sourcing donor material from natural meadows would further decline them. Therefore, we advocate the domestication and mariculture of seagrasses in order to produce the large quantities of seed needed for successful rewilding of the sea with seagrass meadows. We provide a roadmap for our proposed solution and show that 44% of seagrass species have promising reproductive traits for domestication and rewilding by seeds. The principle of partially domesticating species to enable subsequent large-scale rewilding may form a successful shortcut to restore threatened keystone species and their vital ecosystem services.

Seagrass meadows provide multiple global and local ecosystem services, including carbon sequestration (McLeod et al. 2011), coastal protection (James et al. 2021), and water quality improvement (Lamb et al. 2017, Sanchez-Vidal et al. 2021), as well as habitat and fish nursery provision (Nordlund et al. 2016, Unsworth et al. 2019). Seagrass meadows were estimated to have initially occupied over 10% of the total worldwide sea floor that receives sufficient light—that is, 0.6 million square kilometers (Duarte 2017). The global extent of seagrass meadow has, however, almost halved over the last century because of anthropogenic pressures (Waycott et al. 2009). In terms of climate change mitigation alone, this loss implies a missed opportunity of global carbon dioxide (CO2) sequestration, because these lost meadows would have provided approximately 3.6%–8.4% of the CO2 sequestration required by 2030 to get on track toward the Paris targets of limiting global warming to 1.5–2 degrees Celsius (UNEP 2019; for calculations, see the supplemental materials).

## Why we cannot rely on natural recovery alone

Unlike bare land turning green after rain, bare coastal sediments generally do not become vegetated by rooting plants after a favorable turn of the tides. Even when anthropogenic pressures, such as eutrophication are relieved, recolonization by seagrasses typically takes decades to centuries. This may be caused by three key hurdles that rooted plants face in bare shallow coastal environments: high stress levels (e.g., sediment instability), high stochasticity (e.g., water dynamics, storms), and natural fragmentation (e.g., rocky areas, deeper areas, dynamic areas or turbid river mouths). Seagrasses cope with these hurdles by the power of large numbers: large shoot numbers to reach densities that reduce the impact of physical stress and small-scale stochasticity, large meadow areas to generate positive feedback loops at landscape scale, and large numbers of surrounding meadows with dispersing seeds to recolonize meadows lost in large-scale stochastic events. From population dynamic theory, it is known that this combination of strong positive feedback loops and stochasticity inevitably leads to intermittent local population extinctions, meaning that persistence in an area depends on reintroductions from nearby populations within the same metapopulation (Dennis et al. 2016).

Several observations demonstrate the principle that seagrasses persist in their stressful and unpredictable environment through large numbers and through metapopulation dynamics. In the first place, seagrass meadows have been shown to be maintained by positive feedback loops, depending on large numbers or areas, at local scale (e.g., sediment stabilization) and landscape scale (e.g., water clarification; Maxwell et al. 2017). Second, many seagrass species have both effective local and long-distance seed dispersal (Kendrick et al. 2012, McMahon et al. 2014), and isolated populations tend to vanish (Aloitaibi et al. 2019), showing both the potential and operation of metapopulation dynamics. Third, a global analysis has shown that large-scale restoration trials are on average more successful than small-scale trials (van Katwijk et al. 2016). Finally, in multisite, multiyear restoration programs, it is often observed that only a few trials within the program expand vigorously, whereas the remaining trials fail (Suykerbuyk et al. 2016, Paulo et al. 2019, McDonald et al. 2020). These failures can often be explained in hindsight (McDonald et al. 2020) or, at least, alluded to (Suykerbuyk et al. 2016, Paulo et al. 2019), but they cannot be predicted, suggesting the operation of chance dynamics in the recovery process.

On the basis of the observed population dynamics and previous restorations, we therefore believe that successful seagrass recovery requires a large supply of seeds over sustained periods of time and that natural recovery cannot be expected within management-relevant time scales. The most successful case of seagrass restoration worldwide illustrates this principle. The Virginia Bays area on the US East Coast showed no recovery of the seagrass species *Zostera marina* after a disease had driven it extinct in the 1930s. Only, by the 1990s, two new patches had arrived (Orth et al. 2012). Ten years of repeated large-scale seeding then led to the restoration of 17 km2 in 2010, which was estimated to have accelerated the recovery process from an estimated 40 years to only 10 years (Reynolds et al. 2016).

## Domestication for rewilding?

Restoration should be sustainable to fully gain the associated ecosystem services. This may be achieved through so-called rewilding, which is a type of restoration that aims at self-sustainability, thereby reinstating natural dynamic processes in coastal zones (Perino et al. 2019). Although the phrase “rewilding the sea” might yield associations with enhancing megafauna, because these are involved in the most iconic terrestrial rewilding programs, the recent and broader description of rewilding by Perino and coworkers (2019) accurately fits with our plea for rewilding the sea with seagrass: reintroducing seagrasses where they were lost and beyond and recreating a self-sustaining system. The connotation with enhancing megafauna is, however, not lost, because seagrass meadows host several iconic megaherbivores, including dugongs, manatees, and sea turtles. The term *rewilding* also hints at the wildness of the seagrass meadows and their requirement of a large “territory” for self-maintenance. Using this term may therefore enhance awareness that “wild” processes govern the successes and failures of seagrass recovery.

Rewilding the sea with seagrass meadows inherently requires large amounts of donor material, which should preferably not be sourced from natural meadows but could instead be cultured. Paradoxically, *rewilding* the seas with seagrasses could therefore depend on the *domestication* of seagrass. Domestication is the intermediate step between resource management and agriculture (Zeder 2015). It involves a sustained multigenerational, mutualistic relationship in which one organism (i.e., humans) assumes a significant degree of influence over the reproduction and care of another organism (i.e., seagrass) in order to secure a more predictable supply of a resource of interest (i.e., seagrass seed), as was defined by Zeder (2015), where she added, “and through which the partner organism gains advantage over individuals that remain outside this relationship, thereby benefitting and often increasing the fitness of both the domesticator and the target domesticate.” Domestication often leads to changes in traits of the target domesticate, which may be preferred or not preferred. Trait changes can be influenced by adapting selection processes, with or without the use of genomic techniques.

## Advancing from traditional restoration toward rewilding

**Figure 1.**
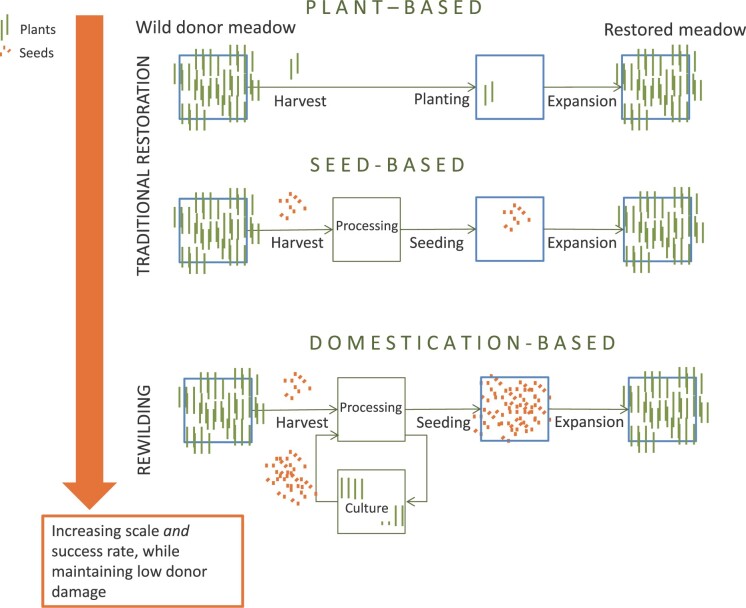
Evolving from plant-based restoration to seed-based restoration to domestication-based rewilding allows for upscaling of the restoration while maintaining low donor damage. With increasing scale, the success rate increases more than linearly (van Katwijk et al. 2016).

Traditional restoration involves the harvesting of donor seeds or plants and subsequent seeding or planting (figure 1). Plant-based restoration involves translocation and planting; seed-based restoration requires an additional processing step of seed extraction and storage. This latter processing step allows for treatments like disinfection, removing invasive species, dormancy breakage, and seed coating (figure 1; Kettenring and Tarsa 2020, Tan et al. 2020). Several technological options may improve success in the different phases of traditional restoration, depending on the environmental context (table 1; van Katwijk et al. 2016, Orth et al. 2020, Tan et al. 2020). Domestication-based restoration adds a cultivation step using mariculture (figure 1). Mariculture is the provisioning system for domestication, providing the infrastructure and techniques to sow, plant, grow, and collect seeds or plants. Going from plant-based to seed-based restoration to domestication-based rewilding, the scale of the restoration effort increases while maintaining low donor damage. The probability of success increases, because larger scales accelerate survival and expansion rates (van Katwijk et al. 2016). Mariculture could also lead to the production of vegetative fragments to be planted for rewilding (not shown in figure 1).

**Table 1.** Technological options employed in different phases of traditional restoration (van Katwijk et al. 2016, Orth et al. 2020, Tan et al. 2020).

Phase of restoration	Technical option
Harvest	Manual or mechanical. Mechanical collection usually involves the excavation of sods. Intertidal (challenging in mud) or subtidal (scuba diving or underwater machines).
Plant material	Rhizome fragments with shoots (or “turions”) Sods: intact units of native sediment with roots, rhizomes and leaves (including plugs or turf pots) Seedlings Seeds
Seed processing	Disinfection, removing invasive species, dormancy breakage, and seed coating
Seed storage	Storage optimization of temperature, water circulation etcetera. Seed losses during storage should trade-off seed losses *in situ* during the unfavorable season^b^
Local habitat treatments, usually temporary	Sediment stabilization (may include reduction of bioturbation; e.g., application of shells or biodegradable structures) Protection against grazing (e.g., exclosures) Wave reduction devices like dams, ridges or exclosures Fertilizer, growth hormone or iron additions
Planting or seeding design	The clustering or spreading of the plants or seeds should trade-off the expected benefits from positive feedback (clustering) and countering natural variability (bat hatching by spreading)
Planting techniques	Anchoring by staples (including rods, bamboos, pegs, sprigs, iron nails or washers), frames (nonweighted; plant material is attached to devices like frames, grids, quadrats, nets, mats or meshes), or weights (provided by rocks, shells, bricks, sandbags or by using weighted frames; TERFS)
Seeding techniques	Broadcasting for example from a boat or while wading (low water level) or walking (intertidal) Buoy-deployed seeding: natural broadcasting from bags with seed-bearing shoots attached to buoys^a^ Injection into the sediment by an automated device or manual injection using dispensers

*Note:* Large-scale habitat improvement may include a reduction of nutrient or sediment loads (e.g., de los Santos et al. 2019, Greening et al. 2018); foodweb restoration (e.g., in the Baltic Sea), a reduction of mesopredators, or an introduction of predators (e.g., Östman et al. 2016); or the restoration of water circulation (Kruk-Dowgiallo 1991, Lenzi et al. 2003). Abbreviation: TERFS, transplanting eelgrass remotely with frame system (Short et al. 2002). ^a^Pickerell and colleagues (2005). ^b^Infantes and colleagues (2016).

**Figure 2.**
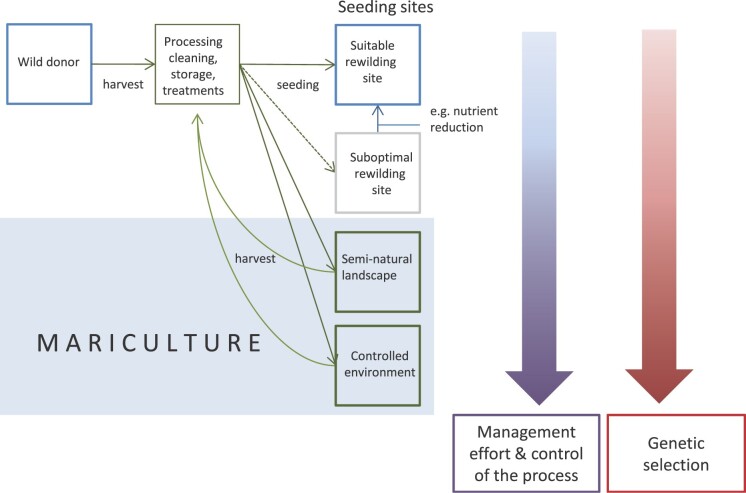
Four types of seeding sites can be distinguished: First, suitable rewilding sites, which are relatively rare (see text). Second, suboptimal rewilding sites, which should be optimized prior or simultaneously with the seeding—for example, by nutrient reduction, removal of physical disturbance or improving the food webs (table 1). Third, for mariculture of seagrass, seminatural landscapes such as abandoned aquacultures can be used or created to allow for frequent harvesting. Fourth, the seagrass plants can be placed in a controlled environment—for instance, in mesocosms or tanks. In concordance, management effort increases toward the totally controlled environment, but also the control of the process increases. In the same direction, genetic selection increases, which can be unwanted, or in some cases perhaps be wanted, see text. Note that on sea level rise in the twenty-first century, subsiding low-lying land may become available for seagrass rewilding or mariculture sites as well.

The mariculture of seagrasses can take place under controlled conditions, in indoor or outdoor tanks or mesocosms. Alternatively, mariculture can be established in semicontrolled conditions—for example, by creating or using seminatural landscapes that meet seagrass habitat criteria (figure 2). These could, for instance, include modified shallow water bodies or newly subsided areas under sea level rise. In general, most seagrasses prefer relatively sheltered conditions, with good water circulation and some nutrient supply (but not too much to avoid algal nuisance), as well as enough light and a sufficiently low frequency of extreme events. Several small-scale mariculture experiments have been performed with a number of seagrass species showing the potential to culture seeds from seed in mesocosms (e.g., Balestri and Lardicci 2012, Tan et al. 2020). However, upscaling seagrass mariculture will require additional research and major financial investment.

For the subsequent rewilding step, determining optimal locations is challenging, because abiotic and biotic environmental conditions change and interact and are therefore partly unpredictable (Suykerbuyk et al. 2016). Locations that have optimal growing conditions for seagrass but are currently not vegetated are thought to be rare. These could include some new sites (e.g., when the depth becomes suitable after natural or human induced sedimentation or when land subsides rise following sea level rise due to climate change) or former seagrass habitats that lost seagrasses because of an incidental catastrophe (e.g., Virginia Bays; Orth et al. 2020). Suboptimal locations, where one or several environmental conditions hamper seagrass recovery, are likely more abundant in our present world. These could for example include areas that lost seagrasses because of eutrophication. Impressive natural recovery has occurred in such areas once the habitat had been improved, provided the areas or surroundings still harbored plentiful remnant seagrasses (de los Santos et al. 2019 and the references therein). This suggests that rehabilitation of environmentally degraded areas—and, potentially, also of newly created coastal inundated areas—could provide fruitful locations for rewilding the sea with seagrass.

## Genetic selection: Wanted and unwanted

Genetic selection would likely occur during the various phases of seagrass mariculture (Zeder 2015, Espeland et al. 2017). Basic restoration guidelines prescribe that donor populations are adapted to a similar environment as the target rewilding area, and have sufficiently high genetic variability to allow for adaptation to environmental change and to avoid inbreeding. In view of this, genetic selection should be minimized (Espeland et al. 2017, Pedrini et al. 2020). If the environment at the mariculture deviates much from that at the donor site, then the probability and effects of genetic selection are expected to be more severe (the red arrow in figure 2) and even more effort may be required to minimize this genetic selection. In terrestrial native seed production programs, guidelines to minimize genetic selection prescribe multiple collections through time and space, sampling of a large genetic variation, promotion of gene flow, reduction of selection and provenance tracking (Espeland et al. 2017, Pedrini et al. 2020).

Genetic selection focusing on certain desirable traits has, to our knowledge, only recently started to become considered in scientific restoration literature, particularly in the context of climate change (van Oppen et al. 2015, Coleman and Goold 2019, Gaitan-Espitia and Hobday 2021, Pazzaglia et al. 2021). Genetic selection for certain traits can be desirable to enhance success in environmentally altered environments. In seagrasses, an example would be the trait for heat resistance in areas such as Chesapeake Bay, Florida, in the United States, and Shark Bay, in Australia, which have regularly experienced lethal summer heat or heat waves in recent years (Lefcheck et al. 2017, Carlson et al. 2018, Strydom et al. 2020). Donor material could be sourced from populations experiencing environmental conditions as projected in the near future for the transplantations site (Pazzaglia et al. 2021). Selection may also focus on preferred traits to deliver ecosystem services or ecosystem goods, such as a large biomass or high seed production. In such a trajectory, we can learn from new domestications on land. In the marine realm, plant domestication is still relatively new (e.g., macroalgae for human consumption) compared with terrestrial domestications, which started millennia ago (Duarte et al. 2007) and were mainly aimed at producing food.

Similar to our aims, current new crop domestications on land are aimed at more resilience and less dependence on human assistance, such as fertilizer additions, and also at enhanced ecosystem services (e.g., perennial crops to prevent soil erosion and sequester carbon; DeHaan et al. 2020, Fernie and Yan 2020). Therefore, new crop domestication is aimed at multiple traits and involves an ideotype breeding approach, which means that breeding should select for desirable characteristics rather than select against defective traits (Tork et al. 2019, Fernie and Yan 2020). Where crop domesticators aim at “ideocrops” in this way (Fernie and Yan 2020), in coral science, the appealing but subjective term *super coral* has emerged to describe corals with superior survivorship (Camp et al. 2018). For coral and kelp restoration in a changing world, the possibilities of assisted evolution have been reviewed, involving for example selective breeding, assisted gene flow, conditioning or epigenetic programming, genetic engineering, and the manipulation of the microbiome (van Oppen et al. 2015, Coleman and Goold 2019), although this approach is much debated (Filbee-Dexter and Smajdor 2019, Coleman et al. 2020, Gaitan-Espitia and Hobday 2021). In short, expert fields of rewilding and domestication are much closer together than in the past, and both wanted and unwanted genetic selection concerns all, allowing for mutual learning.

## Suitable seagrass species traits for domestication and for rewilding

**Figure 3.**
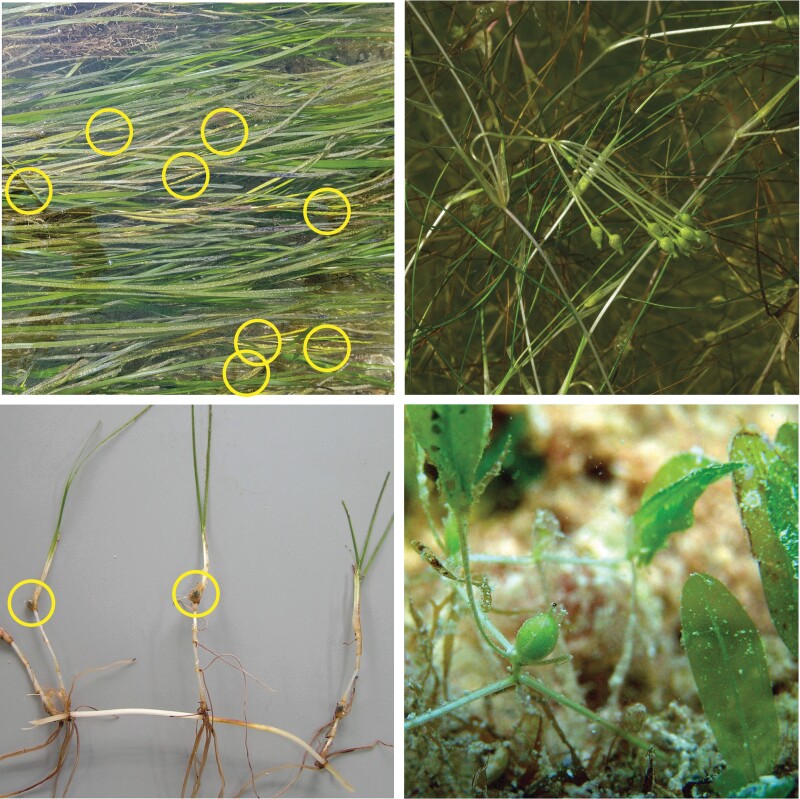
From the top left clockwise four species with high seed production representing four genera: Zostera marina with seeds, Ruppia maritima with seeds, Halophila decipiens with a fruit containing approximately 30 seeds, Halodule wrightii with mature pairs of fruits. Photographs: Marieke van Katwijk, Stephan Mifsad (www.maltawildplants.com) and Brigitta van Tussenbroek. The top right photograph (Ruppia maritima) is reproduced with permission from Stephen Mifsud.

Seeds are central to the proposed rewilding strategy, because they are more donor friendly than vegetative parts, form an easier starting point for breeding and result in faster expansion than clonal growth (Orth et al. 2020). Exploration of suitable seagrass species for domestication and rewilding should therefore start with exploring sexual reproductive traits (figure 3). Most important for the domestication of seagrass species are large seed production potential (i.e., the yield) and harvestability—that is, aboveground seed production that allows harvesting without damaging the (in some cases, long lived) plants. Important traits for rewilding are large seed production potential to allow for rapid population growth and expansion; short life cycle period, which is the period the species can potentially grow from seed and sexually reproduce; and high dispersal potential. We assume in the present article that seeds can disperse when seeds are located aboveground (similar to harvestability).

**Figure 4.**
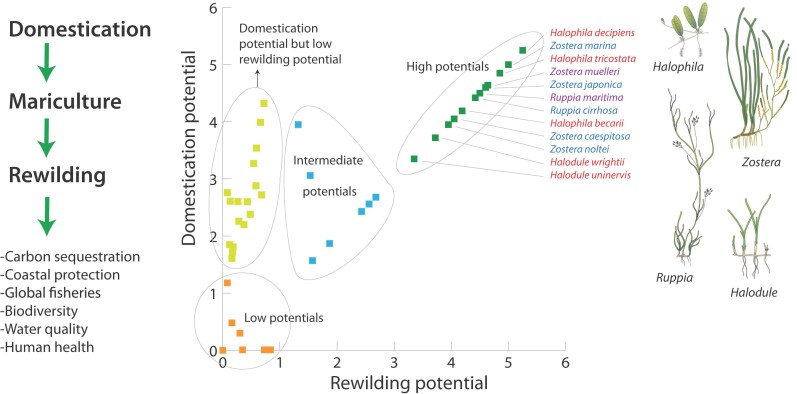
Domestication of seagrass for rewilding the sea. 19 seagrass species (of 43) have a high or intermediate potential for both domestication and rewilding. Domestication potential of seagrass species is derived from maximum seed production and harvestability. Rewilding potential of seagrass species is derived from maximum seed production and life cycle period (see the supplemental materials). The colouring of the species names refers to their global distribution: red, tropical; blue, temperate; purple, both tropical and temperate. Source: The seagrass drawings are reproduced courtesy of the Integration and Application Network, University of Maryland Center for Environmental Science (ian.umces.edu/symbols; except the drawing of Zostera marina).

We applied these criteria on 43 species of seagrass (out of the global 62 species) for which sufficient data was available (a detailed methodology is provided in the supplemental materials). This resulted in 12 species that are highly suitable potential candidates for domestication and rewilding and 7 that are intermediately suitable (figure 4). This means that 44% of the seagrass species have potential for a domestication and rewilding trajectory, and this includes tropical and temperate species (figure 4). Note that many populations of these 12 species, although they have a high *potential* seed production, may produce none or few seeds in some instances or may produce seeds at irregular interannual intervals. The manipulation of seed production would require further research.

Importantly, it should be realized that most of the 12 species have colonizing or opportunistic traits and may provide less substantial and diverse ecosystem services than the larger and more persistent species (e.g., Nordlund et al. 2016). Domestication and rewilding of large climax species may also be targeted but likely requires more time. In most instances, their recovery will also be facilitated by the colonizing or opportunistic species (e.g., Maxwell et al. 2017; an exception being the nonnative Halophila replacing Caribbean climax species under certain conditions, Willette et al. 2014).

## Perspectives for seagrass mariculture

Seagrass mariculture lots may provide some of the same ecosystem services as “wild” seagrass meadows, as long as harvesting of the seeds does not destruct the lots. Interestingly, seagrass mariculture may also provide food. For centuries, seeds of the seagrass species *Z. marina* formed the staple food for the people of Sinaloa, Mexico (Felger and Moser 1973). This seagrass can provide more than 5 tons edible grains per hectare and has a wide global range. Producers of seabread may in fact have a much more favorable starting crop than the terrestrial grains encountered by early farmers 12,000 years ago (supplemental data S1). In general, multiple goods, including medicine, are provided by seagrass, and new applications continue to be discovered (de los Santos et al. 2020).

Seagrass domestication and mariculture, as well as rewilding of the sea with seagrasses at an industrial scale, are perhaps not feasible in 2021. However, it may become a realistic option within decades when climate measures become more stringent. At the same time, low lying countries, facing 0.5 meters or more sea level rise by 2100 (Brown et al. 2018), will have to, voluntarily or involuntarily, give up land for sea. Potential seagrass rewilding areas and mariculture lots may become increasingly available as present agricultural land may subside under sea level rise during the next century (Brown et al. 2018). Established seagrass meadows may subsequently help to prevent further subsidence (James et al. 2021). Note that the seagrass rewilding sites need environmental protection, but they also need to be economically and socioculturally sustainable (Unsworth et al. 2019, as is also reflected in the Sustainable Development Goals of the UN 2017). Financial and legal incentives can facilitate a transition of degrading practices toward more seagrass-friendly use of coastal ground and fuel sustainable innovations (Guerry et al. 2015, UNEP 2020). Locations off site from coastal areas or offshore may be considered for mariculture, although this will involve extra logistic challenges and costs.

Reserving some decades to (start) preparing for domestication and mariculture is not much, given that land-based agriculture has already a history of thousands of years and agricultural techniques still improving. Although seagrass restoration with return of nearly all functions can be achieved within 10–20 years (Orth et al. 2020), preceding monitoring of the habitat characteristics and research into the requirements for seagrasses may take a decade as well. Breeding is an iterative process (Tork et al. 2019, van Tassel et al. 2020) and may take an additional 8–20 generations to show phenotypic differences (DeHaan et al. 2020, Fernie and Yan 2020). Note that full return of ecosystem services may require more time than the 10–20 years reported in Orth and colleagues (2020): In many parts of the world, these services are generated by species with less suitable domestication and rewilding traits (located roughly in the eastern hemisphere and the wider Caribbean tropics)—that is, larger climax species (Nordlund et al. 2016). Rewilding with one of the 12 suitable species may help to recover these climax species through successional pathways.

In conclusion, domestication to enable rewilding could become an important strategy to restore keystone species in a changing world, shaping valuable ecosystems and their services and goods. For seagrasses, a domestication trajectory could start with the species with high seed production and short life cycles selected in this review. The proposed domestication of seagrass to enable rewilding may likely exceed budgets traditionally assigned to nature restoration projects by orders of magnitude but will also be more profitable. Rather, investments should be part of budgets required for climate change mitigation, agricultural innovations, and land and sea use transitions in the future era.

## Supplementary Material

biab092_Supplemental_Files

## Author Biographical

Marieke M. van Katwijk (m.vankatwijk@science.ru.nl), Steef V. Hanssen, and A. Jan Hendriks are affiliated with the Department of Environmental Science, Institute for Water and Wetland Research, at Radboud University, in Nijmegen, The Netherlands. Brigitta I. van Tussenbroek is affiliated with the Reef Systems Unit at the Instituto de Ciencias del Mar y Limnología, at the Universidad Nacional Autónoma de México, in Puerto Morelos, Quintana Roo, Mexico. Lucien Hanssen is affiliated with Deining Sustainable Coastal Zone Management, in Nijmegen, The Netherlands.

## References

[bib1] Alotaibi NM , Kenyon EJ, Cook KJ, Borger L, Bull JC. 2019. Low genotypic diversity and long-term ecological decline in a spatially structured seagrass population. Scientific Reports 9: 18387. 31804557 10.1038/s41598-019-54828-1 PMC6895181

[bib2] Balestri E , Lardicci C. 2012. Nursery-propagated plants from seed: A novel tool to improve the effectiveness and sustainability of seagrass restoration. Journal of Applied Ecology 49: 1426–1435.

[bib3] Brown S , Nicholls RJ, Goodwin P, Haigh ID, Lincke D, Vafeidis AT, Hinkel J. 2018. Quantifying land and people exposed to sea-level rise with no mitigation and 1.5 degrees C and 2.0 degrees C rise in global temperatures to year 2300. Earths Future 6: 583–600.

[bib4] Camp EF , Schoepf V, Suggett DJ. 2018. How can “Super Corals” facilitate global coral reef survival under rapid environmental and climatic change? Global Change Biology 24: 2755–2757. 29582529 10.1111/gcb.14153

[bib5] Carlson DF , Yarbro LA, Scolaro S, Poniatowski M, McGee-Absten V, Carlson PR. 2018. “Sea surface temperatures and seagrass mortality in Florida Bay: Spatial and temporal patterns discerned from MODIS and AVHRR data.” Remote Sensing of Environment 208: 171–188.

[bib6] Coleman MA , Goold HD. 2019. Harnessing synthetic biology for kelp forest conservation. Journal of Phycology 55: 745–751. 31152453 10.1111/jpy.12888

[bib7] Coleman MA et al. 2020. Restore or redefine: future trajectories for restoration. Frontiers in Marine Science 7: 237.

[bib8] de los Santos CB et al. 2019. Recent trend reversal for declining European seagrass meadows. Nature Communications 10: 3356. 10.1038/s41467-019-11340-4 PMC6659699 31350407

[bib9] de los Santos CB et al. 2020. Seagrass ecosystem services: Assessment and scale of benefits. Pages 20–35 in Out of the Blue: The Value of Seagrasses to the Environment and to People. UN Environment Programme.

[bib10] DeHaan L , Larson S, Lopez-Marques RL, Wenkel S, Gao CX, Palmgren M. 2020. “Roadmap for accelerated domestication of an emerging perennial grain crop.” Trends in Plant Science 25: 525–537. 32407693 10.1016/j.tplants.2020.02.004

[bib11] Dennis B , Assas L, Elaydi S, Kwessi E, Livadiotis G. 2016. Allee effects and resilience in stochastic populations. Theoretical Ecology 9: 323–335.

[bib12] Duarte CM. 2017. Reviews and syntheses: Hidden forests, the role of vegetated coastal habitats in the ocean carbon budget. Biogeosciences 14: 301–310.

[bib13] Duarte CM , Marbà N, Holmer M. 2007. Rapid domestication of marine species. Science 316: 382–383. 17446380 10.1126/science.1138042

[bib14] Espeland EK , Emery NC, Mercer KL, Woolbright SA, Kettenring KM, Gepts P, Etterson JR. 2017. Evolution of plant materials for ecological restoration: Insights from the applied and basic literature. Journal of Applied Ecology 54: 102–115.

[bib15] Felger R , Moser MB. 1973. Eelgrass (*Zostera marina* L) in Gulf of California: Discovery of its nutritional-value by Seri Indians. Science 181: 355–356. 17832031 10.1126/science.181.4097.355

[bib16] Fernie AR , Yan JB. 2020. Targeting key genes to tailor old and new crops for a greener agriculture. Molecular Plant 13: 354–356. 32074483 10.1016/j.molp.2020.02.007

[bib17] Filbee-Dexter K , Smajdor A. 2019. Ethics of assisted evolution in marine conservation. Frontiers in Marine Science 6: 20.

[bib18] Gaitan-Espitia JD , Hobday AJ. 2021. Evolutionary principles and genetic considerations for guiding conservation interventions under climate change. Global Change Biology 27: 475–488. 32979891 10.1111/gcb.15359

[bib19] Guerry AD et al. 2015. Natural capital and ecosystem services informing decisions: From promise to practice. Proceedings of the National Academy of Sciences 112: 7348–7355. 10.1073/pnas.1503751112 PMC4475956 26082539

[bib20] Infantes E , Eriander L, Moksnes PO. 2016. Eelgrass (*Zostera marina*) restoration on the west coast of Sweden using seeds. Marine Ecology Progress-Series 546: 31–45.

[bib21] James RK et al. 2021. Tropical biogeomorphic seagrass landscapes for coastal protection: Persistence and wave attenuation during major storms events. Ecosystems 24: 301–318.

[bib22] Kendrick GA et al. 2012. The central role of dispersal in the maintenance and persistence of seagrass populations. BioScience 62: 56–65.

[bib23] Kettenring KM , Tarsa EE. 2020. Need to seed? Ecological, genetic, and evolutionary keys to seed-based wetland restoration. Frontiers in Environmental Science 8: 109.

[bib24] Kruk-Dowgiallo L. 1991. Long-term changes in the structure of underwater meadows of the Puck Lagoon. Acta Ichthyologica et Piscatoria 21: 77–84.

[bib25] Lamb JB , van de Water J, Bourne DG, Altier C, Hein MY, Fiorenza EA, Abu N, Jompa J, Harvell CD. 2017. Seagrass ecosystems reduce exposure to bacterial pathogens of humans, fishes, and invertebrates. Science 355: 731–733. 28209895 10.1126/science.aal1956

[bib26] Lefcheck JS , Wilcox DJ, Murphy RR, Marion SR, Orth RJ. 2017. Multiple stressors threaten the imperiled coastal foundation species eelgrass (*Zostera marina*) in Chesapeake Bay, USA. Global Change Biology 23: 3474–3483. 28165203 10.1111/gcb.13623

[bib27] Lenzi M , Palmieri R, Porrello S. 2003. Restoration of the eutrophic Orbetello lagoon (Tyrrhenian Sea, Italy): Water quality management. Marine Pollution Bulletin 46: 1540–1548. 14643780 10.1016/S0025-326X(03)00315-1

[bib28] Maxwell PS et al. 2017 . The fundamental role of ecological feedback mechanisms for the adaptive management of seagrass ecosystems: A review. Biological Reviews 92: 1521–1538. 27581168 10.1111/brv.12294

[bib29] McDonald AM , Christiaen B, Major KM, Cebrian J. 2020. The influence of seagrass donor source on small-scale transplant resilience. Aquatic Conservation-Marine and Freshwater Ecosystems 30: 730–742.

[bib30] McLeod E , Chmura GL, Bouillon S, Salm R, Bjork M, Duarte CM, Lovelock CE, Schlesinger WH, Silliman BR. 2011. A blueprint for blue carbon: Toward an improved understanding of the role of vegetated coastal habitats in sequestering CO_2_. Frontiers in Ecology and the Environment 9: 552–560.

[bib31] McMahon K et al. 2014 . The movement ecology of seagrasses. Proceedings of the Royal Society B 281: 20140878. 25297859 10.1098/rspb.2014.0878 PMC4213608

[bib32] Nordlund LM , Koch EW, Barbier EB, Creed JC. 2016. Seagrass Ecosystem Services and Their Variability across Genera and Geographical Regions. PLOS ONE 11: e0163091. 27732600 10.1371/journal.pone.0163091 PMC5061329

[bib33] Orth RJ , Moore KA, Marion SR, Wilcox DJ, Parrish DB. 2012. Seed addition facilitates eelgrass recovery in a coastal bay system. Marine Ecology Progress Series 448: 177–195.

[bib34] Orth RJ , Lefcheck JS, McGlathery KS, Aoki L, Luckenbach MW, Moore KA, Oreska MPJ, Snyder R, Wilcox DJ, Lusk B. 2020. Restoration of seagrass habitat leads to rapid recovery of coastal ecosystem services. Science Advances 6: eabc6434. 33028530 10.1126/sciadv.abc6434 PMC7541073

[bib35] Östman O , Eklöf J, Eriksson BK, Olsson J, Moksnes PO, Bergström U. 2016. Top-down control as important as nutrient enrichment for eutrophication effects in North Atlantic coastal ecosystems. Journal of Applied Ecology 53: 1138–1147.

[bib36] Paulo D , Cunha AH, Boavida J, Serrao EA, Goncalves EJ, Fonseca M. 2019. Open coast seagrass restoration. Can we do it? Large scale seagrass transplants. Frontiers in Marine Science 6: 52.

[bib37] Pazzaglia J , Nguyen HM, Santillan-Sarmiento A, Ruocco M, Dattolo E, Marín-Guirao L, Procaccini G. 2021. The genetic component of seagrass restoration: What we know and the way forwards. Water 13: 829.

[bib38] Pedrini S et al. 2020 . Collection and production of native seeds for ecological restoration. Restoration Ecology 28: S228–S238.

[bib39] Perino A et al. 2019. Rewilding complex ecosystems. Science 364: eaav5570. 31023897 10.1126/science.aav5570

[bib40] Pickerell CH , Schott S, Wyllie-Echeverria S. 2005. Buoy-deployed seeding: Demonstration of a new eelgrass (*Zostera marina* L.) planting method. Ecological Engineering 25: 127–136.

[bib41] Reynolds LK , Waycott M, McGlathery KJ, Orth RJ. 2016. Ecosystem services returned through seagrass restoration. Restoration Ecology 24: 583–588.

[bib42] Sanchez-Vidal A , Canals M, de Haan WP, Romero J, Veny M. 2021. Seagrasses provide a novel ecosystem service by trapping marine plastics. Scientific Reports 11: 254. 33446674 10.1038/s41598-020-79370-3 PMC7809288

[bib43] Short FT , Kopp BS, Gaeckle J, Tamaki H. 2002. Seagrass ecology and estuarine mitigation: A low-cost method for eelgrass restoration. Japan Fisheries Science 68: 1759–1762.

[bib44] Strydom SK et al. 2020 . Too hot to handle: Unprecedented seagrass death driven by marine heatwave in a world heritage area. Global Change Biology 26: 3525–3538. 32129909 10.1111/gcb.15065

[bib45] Suykerbuyk W , Govers LL, Bouma TJ, Giesen W, de Jong DJ, van de Voort R, Giesen K, Giesen PT, van Katwijk MM. 2016. Unpredictability in seagrass restoration: Analysing the role of positive feedback and environmental stress on *Zostera noltii* transplants. Journal of Applied Ecology 53: 774–784.

[bib46] Tan YM et al. 2020 . Seagrass restoration is possible: insights and lessons from Australia and New Zealand. Frontiers in Marine Science 7: 617.

[bib47] Tork DG , Anderson NO, Wyse DL, Betts KJ. 2019. Domestication of perennial flax using an ideotype approach for oilseed, cut flower, and garden performance. Agronomy-Basel 9: 707.

[bib48] [UN] United Nations . 2017. Global Indicator Framework for the Sustainable Development Goals and Targets of the 2030 Agenda for Sustainable Development United Nations A/RES/71/313.

[bib49] [UNEP] United Nations Environment Programme . 2019. Emissions Gap Report 2019: Executive Summary. UNEP.

[bib50] [UNEP] United Nations Environment Programme . 2020. Out of the Blue: The Value of Seagrasses to the Environment and to People. World Seagrass Association.

[bib51] Unsworth RKF , Nordlund LM, Cullen-Unsworth LC. 2019. Seagrass meadows support global fisheries production. Conservation Letters 12: e12566.

[bib52] van Katwijk MM et al. 2016. Global analysis of seagrass restoration: The importance of large-scale planting. Journal of Applied Ecology 53: 567–578.

[bib53] van Oppen MJH , Oliver JK, Putnam HM, Gates RD. 2015. Building coral reef resilience through assisted evolution. Proceedings of the National Academy of Sciences 112: 2307–2313. 10.1073/pnas.1422301112 PMC4345611 25646461

[bib54] Van Tassel DL , Tesdell O, Schlautman B, Rubin MJ, DeHaan LR, Crews TE, Krug AS. 2020. New Food crop domestication in the age of gene editing: Genetic, agronomic and cultural change remain co-evolutionarily entangled. Frontiers in Plant Science 11: 789. 32595676 10.3389/fpls.2020.00789 PMC7300247

[bib55] Waycott M et al. 2009. Accelerating loss of seagrasses across the globe threatens coastal ecosystems. Proceedings of the National Academy of Sciences 106: 12377–12381. www.pnas.org/cgi/https://doi.org/10.1073/pnas.0905620106. 10.1073/pnas.0905620106 PMC2707273 19587236

[bib56] Willette DA , Chalifour J, Debrot AOD, Engel MS, Miller J, Oxenford HA, Short FT, Steiner SCC, Vedie F. 2014. Continued expansion of the trans-Atlantic invasive marine angiosperm *Halophila stipulacea* in the Eastern Caribbean. Aquatic Botany 112: 98–102.

[bib57] Zeder MA. 2015. Core questions in domestication research. Proceedings of the National Academy of Sciences 112: 3191–3198. 10.1073/pnas.1501711112 PMC4371924 25713127

