# Supplementary material for: Rewilding the Sea with Domesticated Seagrass
Source: Bioscience. 2021 Sep 22;71(11):1171–8. doi: 10.1093/biosci/biab092 (PMC8560307; doi:10.1093/biosci/biab092)
Supplement: biab092_Supplemental_Files [file biab092_supplemental_files.zip › Data_S1.pdf]

SHEET 1

Species	Tropical /Temperate	Potential seed production [seeds $m^{-2} y^{-1}$]	Aboveground seed release	Life cycle period [y]	Harvestable	Domestication	Rewilding	Group	
Halodule uninervis	tropical	2218	no	1	yes	3.35	3.35	1	
Halodule wrightii	tropical	5200	no	1	yes	3.72	3.72	1	
Halophila becarii	tropical	15608	yes	1	yes	4.19	4.19	1	
Halophila decipiens	tropical	176880	yes	1	yes	5.25	5.25	1	
Halophila tricostata [1]	tropical	70000	yes	1	yes	4.85	4.85	1	
Ruppia cirrhosa	temperate	26242	yes	1	yes	4.42	4.42	1	
Ruppia maritima	both	31809	yes	1	yes	4.50	4.50	1	
Zostera caespitosa	temperate	11151	yes	1	yes	4.05	4.05	1	
Zostera japonica	temperate	40244	yes	1	yes	4.60	4.60	1	
Zostera marina	temperate	100376	yes	1	yes	5.00	5.00	1	
Zostera muelleri	both	43884	yes	1	yes	4.64	4.64	1	
Zostera noltei	temperate	9000	yes	1	yes	3.95	3.95	1	
Halophila engelmanii	tropical	74	yes	1	yes	1.87	1.87	2	
Halophila ovalis	tropical	480	yes	1	yes	2.68	2.68	2	
Halophila stipulacea	tropical	270	yes	1	yes	2.43	2.43	2	
Ruppia tuberosa	tropical	37	yes	1	yes	1.57	1.57	2	
Syringodium filiforme	tropical	9000	yes	3	yes	3.95	1.32	2	
Zostera caulescens	temperate	1144	yes	2	yes	3.06	1.53	2	
Zostera tasmanica	both	360	yes	1	yes	2.56	2.56	2	
Amphibolis antarctica	temperate	400	yes	10	yes	2.60	0.26	3	
Amphibolis griffithii	temperate	52	yes	10	yes	1.72	0.17	3	
Enhalus acoroides	tropical	65	yes	10	yes	1.81	0.18	3	
Phyllospadix iwatensis	temperate	21000	yes	6	yes	4.32	0.72	3	
Phyllospadix japonicus	temperate	3445	yes	6	yes	3.54	0.59	3	
Phyllospadix scouleri	temperate	1846	yes	6	yes	3.27	0.54	3	
Phyllospadix serrulatus	temperate	160	yes	6	yes	2.20	0.37	3	
Phyllospadix torreyi	temperate	9756	yes	6	yes	3.99	0.66	3	
Posidonia australis	temperate	751	yes	5	yes	2.88	0.58	3	
Posidonia coriacea	temperate	70	yes	15	yes	1.85	0.12	3	
Posidonia oceanica	temperate	574	yes	35	yes	2.76	0.08	3	
Posidonia sinuosa	temperate	409	yes	20	yes	2.61	0.13	3	
Thalassia hemprichii	tropical	402	yes	6	yes	2.60	0.43	3	
Thalassia testudinum	tropical	526	yes	4	yes	2.72	0.68	3	
Thalassodendron ciliatum	tropical	181	yes	8	yes	2.26	0.28	3	
Thalassodendron pachyrhizum	temperate	41	yes	10	yes	1.61	0.16	3	
Zostera asiatica	temperate	240	yes	5	yes	2.38	0.48	3	
Cymodocea nodosa	temperate	340	no	3	no	0.00	0.00	4	*
Cymodocea rotundata	tropical	11	no	3	no	0.00	0.00	4	*
Cymodocea serrulata	both	159	no	3	no	0.00	0.00	4	*
Halophila baillonii	tropical	2	yes	1	yes	0.30	0.30	4	
Posidonia ostenfeldii	temperate	15	yes	15	yes	1.18	0.08	4	
Ruppia megacarpa	temperate	1	yes	2	yes	0.00	0.00	4	
Syringodium isoetifolium	tropical	3	yes	3	yes	0.48	0.16	4	

Criteria for grouping	
**Domestication**	
**3**	high
**1.5**	intermediate
**Rewilding**	
**3**	high
**1**	intermediate

*set at zero because of belowground seed release, see supplementary information

	Domest - Rewild
group 1	High - High
group 2	Intermed - Intermed
group 3	Domestication High or Intermed - Rewilding low
group 4	Low - Low

SHEET 2

Family	Species	Life cycle period yr	Above-ground seed-release	Potential seed production	Determination of potential seed production	Maximum number of ovules per reprod unit [O]	Reproductive density (reprod unit m^{-2}) [RD]	Reproductive unit	Determination of reproductive density	Literature references for life cycle period	Literature references for maximum number of ovules per reprod unit	Literature references on other reported reproductive features
Cymodoceaceae	*Amphibolis antarctica*	10	yes	2	reported (seedling density)			gynoecium	400 highest density of seedlings/m2	Short et al. 2011		Rivers et al. 2011
Cymodoceaceae	*Amphibolis griffithii*	10	yes	52	O x RD	2	26.0	gynoecium	788 shoots, 3.3%flowering	Short et al. 2011	Den Hartog 1970,	Marba & Walker 1999
Cymodoceaceae	*Cymodocea angustata*		no	NA				gynoecium				
Cymodoceaceae	*Cymodocea nodosa*	3	no	340	reported			gynoecium		Short et al. 2011		Buia&Mazzella 1991
Cymodoceaceae	*Cymodocea rotundata*	>1	no	11	O x RD	2	5.6	gynoecium	171 shoots, 6.5% flowering, ~50% female?	assumed similar to	Den Hartog 1970	Duarte et al. 1997
Cymodoceaceae	*Cymodocea serrulata*	>1	no	159	reported			gynoecium		assumed similar to		Taylor&Rasheed 2012
Hydrocharitaceae	*Enhalus acoroides*	10	yes	65	O x RD	13	5.0	gynoecium	reported	Short et al. 2011	Den Hartog 1970	Patankar et al. 2019
Cymodoceaceae	*Halodule pinifolia*	NA	no	NA				gynoecium				
Cymodoceaceae	*Halodule uninervis*	1	no	2218	reported (bank)			gynoecium		assumed similar to		Inglis (2000)
Cymodoceaceae	*Halodule wrightii*	1	no	5200	reported			gynoecium		van Tussenbroek		Kowalsky&DeYoe 2016
Hydrocharitaceae	*Halophila capricornii*	NA	yes	NA				gynoecium				
Hydrocharitaceae	*Halophila australis*	NA	yes	NA				gynoecium				
Hydrocharitaceae	*Halophila baillonii*	1	yes	2	O x RD	20	0.1	gynoecium	≤0.1 female flower m^{-2}	Short et al. 2011	Den Hartog 1970	Van Tussenbroek et al. 2010
Hydrocharitaceae	*Halophila becarii*	1	yes	15608	reported			gynoecium		Short et al. 2011		Zakaria et al. 2002
Hydrocharitaceae	*Halophila decipiens*	1	yes	176880	reported			gynoecium		Short et al. 2011		Kuo & Kirkman 1995
Hydrocharitaceae	*Halophila engelmanii*	1	yes	74	reported			gynoecium		McMillan 1987		McMillan 1987
Hydrocharitaceae	*Halophila haiwaiiana*	NA	yes	NA				gynoecium				
Hydrocharitaceae	*Halophila johnsonii*	NA	yes	NA				gynoecium				
Hydrocharitaceae	*Halophila minor*	NA	yes	NA				gynoecium				
Hydrocharitaceae	*Halophila nipponica*	NA	yes	NA				gynoecium				
Hydrocharitaceae	*Halophila ovalis*	1	yes	480	reported (bank)			gynoecium		Angsupanich 1996		Kuo & Kirkman 1992
Hydrocharitaceae	*Halophila ovata*	NA	yes	NA				gynoecium				
Hydrocharitaceae	*Halophila spinulosa*	2	yes	NA				gynoecium		Short et al. 2011		
Hydrocharitaceae	*Halophila stipulacea*	1	yes	270	O x RD	40	6.8	gynoecium	15% branches fem., 0.2 fruits/branch, branch=20 shoots, 4500 shoots m^{-2}	Short et al. 2011	Den Hartog 1970	Malm 2009 (fruits), Lipkin 1979 (shoot density)
Hydrocharitaceae	*Halophila tricostata*	1	yes	70000	reported			gynoecium		Short et al. 2011		Kuo et al. 1993
Zosteraceae	*Phyllospadix iwatensis*	6	yes	21000	O x RD	14	1500.0	spathe	reported	Short et al. 2011	Den Hartog 1970	Yabe et al. 1995
Zosteraceae	*Phyllospadix japonicus*	6	yes	3445	O x RD	11	322.0	spathe	reported	Short et al. 2011	Den Hartog 1970	Park & Lee 2009
Zosteraceae	*Phyllospadix scouleri*	6	yes	1846	O x RD	26	71.0	spathe	reported	Short et al. 2011	Den Hartog 1970	Shelton 2008
Zosteraceae	*Phyllospadix serrulatus*	6	yes	160	O x RD	10	16.0	spathe	reported	Short et al. 2011	Den Hartog 1970	Shelton 2008
Zosteraceae	*Phyllospadix torreyi*	6	yes	9756	reported			spathe		Short et al. 2011		Williams 1995
Posidoniaceae	*Posidonia angustifolia*	5	yes	NA				inflorescence		Short et al. 2011		
Posidoniaceae	*Posidonia australis*	5	yes	751	O x RD	16.4	45.8	inflorescence	reported	Short et al. 2011		Smith & Walker 2002
Posidoniaceae	*Posidonia coriacea*	15	yes	70	reported			inflorescence		Short et al. 2011		Campey et al. 2002
Posidoniaceae	*Posidonia denhartogii*	15	yes	NA				inflorescence		Short et al. 2011		
Posidoniaceae	*Posidonia kirkmanii*	15	yes	NA				inflorescence		Short et al. 2011		
Posidoniaceae	*Posidonia oceanica*	35	yes	574	reported			inflorescence		Short et al. 2011		Balestri&Cinelli 2003
Posidoniaceae	*Posidonia ostenfeldii*	15	yes	15	reported			inflorescence		Short et al. 2011		Waycott et al. 2014
Posidoniaceae	*Posidonia sinuosa*	20	yes	409	O x RD	9.8	41.7	inflorescence	reported	Short et al. 2011		Smith & Walker 2002
Ruppiaceae	*Ruppia cirrhosa*	1	yes	26242	reported			inflorescence		Short et al. 2011		Vromans et al. 2013
Ruppiaceae	*Ruppia maritima*	1	yes	31809	reported (bank)			inflorescence		Short et al. 2011		McGovern 2009
Ruppiaceae	*Ruppia megacarpa*	2	yes	1	reported			inflorescence		Jacobs & Brock		Jacobs & Brock 1982 (almost no
Ruppiaceae	*Ruppia polycarpa*	1	yes	1000	reported (bank)			inflorescence		Short et al. 2011		Vollebergh & Congdon 1986
Ruppiaceae	*Ruppia tuberosa*	1	yes	37	reported (bank)			inflorescence		Short et al. 2011		Porter 2007
Cymodoceaceae	*Syringodium filiforme*	3	yes	9000	reported			gynoecium		Short et al. 2011		McMillan 1981
Cymodoceaceae	*Syringodium isoetifolium*	3	yes	3	O x RD	2	1.7	gynoecium		assumed similar *S.*	Den Hartog 1970	Clores&Agoo 2013
Hydrocharitaceae	*Thalassia hemprichii*	6	yes	402	O x RD	3	134.0	gynoecium		Short et al. 2011	Kuo et al. 1991	Rollon et al 2001
Hydrocharitaceae	*Thalassia testudinum*	4	yes	526	O x RD	6	87.6	gynoecium	730 shoots, ~12% female	Thorhaug 1979		Den Hartog 1970
Cymodoceaceae	*Thalassodendron ciliatum*	8	yes	181	O x RD	2	90.3	gynoecium	140 shoots, 1.29 flowers/shoot, ~ 50% female?	Short et al. 2011	Den Hartog 1970	Kamermans et al. 2001,
Cymodoceaceae	*Thalassodendron pachyrhizur*	10	yes	41	O x RD	2	20.7	gynoecium	667 shoots, 6.2%flowering, ~50% female?	Short et al. 2011	Den Hartog 1970	Marba & Walker 1999
Zosteraceae	*Zostera asiatica*	5	yes	240	O x RD	20	12.0	spathe	reported	Short et al. 2011	Den Hartog 1970	Wanatabe et al. 2005
Zosteraceae	*Zostera caespitosa*	1	yes	11151	O x RD	35	318.6	spathe	9% of 472 shoots m^{-2} with rhipidia (7.5 spathes/rhipidia)	Short et al. 2011	Den Hartog 1970	Lee et al. 2005
Zosteraceae	*Zostera capensis*	4	yes	NA				spathe		Short et al. 2011		
Zosteraceae	*Zostera caulescens*	2	yes	1144	O x RD	13	88.0	spathe	reported	Short et al. 2011	Den Hartog 1970	Kim et al. 2014
Zosteraceae	*Zostera geojeensis*	NA	NA	NA				NA				
Zosteraceae	*Zostera japonica*	1	yes	40244	reported			spathe		Short et al. 2011		Zhang et al. 2020
Zosteraceae	*Zostera marina*	1	yes	100376	reported			spathe		Short et al. 2011		Meling-Lopez&Ibarra-Obando
Zosteraceae	*Zostera muelleri*	1	yes	43884	O x RD	12	3657.0	spathe	1219 shoots m^{-2} with on average 3 spathes	Hamdorf & Kirkman	Den Hartog 1970	Peterken & Conacher (1997)
Zosteraceae	*Zostera nigricaulis*	NA	yes	NA				spathe				
Zosteraceae	*Zostera noltei*	1	yes	9000	reported			spathe		Short et al. 2011		Hootsmans et al. 1987
Zosteraceae	*Zostera pacifica*	NA	NA	NA				NA				
Zosteraceae	*Zostera polychlamys*	NA	no	NA				spathe				
Zosteraceae	*Zostera tasmanica*	1	no	360	reported			spathe		Short et al. 2011		Campey et al. 2002

SHEET 3

**parameter**	**unit**	**description**
Species and Family	descriptive	From list of Short et al. 2011: excluding DD (data deficient) species
Life cycle period	year	The minimum period the species can potentially grow from seed and reproduce: corresponds with generation length in from Short et al. 2011, unless mentioned otherwise
Belowground seed release	category	Yes or no
Potential seed production	no. seeds m^{-2}	Potential reproductive capacity: maximum number of seeds produced. Can be reported or calculated, see next columns
Determination of potential seed production	category	Reported or calculated (by multiplication of ovules per reproductive unit (O) and reproductive density (RD))
Ovules/reproductive unit (O)*	no.	reproductive unit = flower (Hydrocharitaceae), grouped flowers covered by the same by bracts (Cymodoceaceae), spathes (Zosteraceae), spikes (Posidoniaceae) or inflorescences (Ruppiaceae)
Reproductive density (RD)	no. m^{-2}	If necessary, only for calculation of potential seed production: maximal number of (female) flowers m^{-2} (*Zostera* : number of spathes*average number of flowers/spathe), or percentage of flowering shoots (to be multiplied with shoot density, next column). (bank): data are from the seed bank.
Reproductive unit	descriptive	Floral arrangements vary among seagrass genera, from solitary flowers to complex inflorescences. Depending on the arrangements, researchers have determined density of reproductive structures in different manners; mostly based on what was most easily visible and discernable in the field. For the ease of this study we have named such easily defined reproductive structures or groups of reproductive structes as "reproductive unit", which can be a solitary fruit or flower (e.g. *Thalassia*), a single infloresce on a complex inflorescence (e.g. spathe), or an inflorescence (e.g. *Ruppia*).
Determination of reproductive density	descriptive	Explains how reproductive density is determined; reproductive density is either reported, or is calculated from other reported traits as described in the cell.

SHEET 4

Angsupanich, S. 1996. Seagrasses and epiphytes in Thale Sap Songkhla, Southern Thailand. La mer 34: 67-73

Balestri, E. and Cinelli, F., 2003 Sexual reproductive success in *Posidonia oceanica* . Aquatic Botany, 75(1): 21-32.

Buia, M. C., & Mazzella, L. (1991). Reproductive phenology of the Mediterranean seagrasses *Posidonia oceanica* (L.) Delile, *Cymodocea nodosa* (Ucria) Aschers., and *Zostera noltii* Hornem. Aquatic Botany, 40(4), 343-362.

Campey ML, Kendrick GA, Walker DI (2002) Interannual and small-scale spatial variability in sexual reproduction of the seagrasses *Posidonia coriacea* and *Heterozostera tasmanica* , southwestern Australia. Aquatic Botany 74: 287-297

Clores MA, Agoo EM 2013. Flowering distribution and fruiting success of *Syringodium isoetifolium* (Cymodeceaceae) in Bogtong Bay, Lahuy Island, Caramoan, Philippines. Academis Research international 4: 1-33

Den Hartog C (1970) Seagrasses of the world. Verhandelingen de Koninklijke Nederlandse Akadenie van Wetenschappen, North Holland Publishing Company, Amsterdan, London.

Duarte, C.M., Uri, J.S., Agawin, N.S.R., Fortes, M.D., Vermaat, J.E. and Marba, N., 1997. Flowering frequency of Philippine seagrasses. Botanica Marina, 40: 497-500.

Gullström, M., Lunden, B., Bodin, M., Kangwe, J., Ohman, M.C., Mtolera, M.S.P. and Björk, M., 2006. Assessment of changes in the seagrass-dominated submerged vegetation of tropical Chwaka Bay (Zanzibar) using satellite remote sensing. Estuarine Coastal And Shelf Science, 67(3): 399-408.

Hamdorf, I., Kirkman, H. 1995. Status of Australian seagras. In: Hamdorf, I.; Kirkman, H., editor/s. Canberra, A.C.T.: Fisheries Pollution and Marine Environment Committee

Hootsmans M.J.M., Vermaat J.E., Van Vierssen W. (1987) Seedbank development, germination and early seedling survival of two seagrass species from the Netherlands: *Zostera marina* L. and *Zostera noltii* Hornem. Aquat Bot 28: 275-285.

Inglis, G.J., 2000. Disturbance-related heterogeneity in the seed banks of a marine angiosperm. Journal of Ecology, 88: 88-99.

Jacobs SWL, Brock MA (1982). A revision of the genus *Ruppia* (Potamogetonaceae) in Australis. Aquat Bot 14: 325-337.

Kamermans, P., Hemminga, M. A., Marbà, N., Mateo, M. A., Mtolera, M., & Stapel, J. (2001). Leaf production, shoot demography, and flowering of *Thalassodendron ciliatum* along the east African coast. Aquatic Botany, 70, 243-258.

Kim J.H., S.H. Park, Y.K. Kim, J.I. Park, K.S. Lee. 2014. Seasonal growth dynamics of the seagrass *Zostera caulescens* on the eastern coast of Korea. Ocean Science Journal 49:391-402

Kowalski, J. L., & DeYoe, H. R. (2016). Flowering and seed production in the subtropical seagrass, *Halodule wrightii* (shoal grass). *Botanica marina* , *59* (2-3), 193-199.

Kuo, J., Coles, R.G., Long, W.J.L. and Mellors, J.E., 1991. Fruits and seeds of *Thalassia hemprichii* (Hydrocharitaceae) from Queensland, Australia. Aquatic Botany, 40: 165-173.

Kuo, J., Long, W.L. and Coles, R.G., 1993. Occurrence and fruit and seed biology of *Halophila tricostata* Greenway (Hydrocharitaceae). Australian Journal of Marine and Freshwater Research, 44: 43-57.

Kuo, J. and Kirkman, H., 1992. Fruits, seeds and germination in the seagrass *Halophila ovalis* (Hydrocharitaceae). Botanica Marina, 35: 197-204.

Kuo, J, H. Kirkman (1995) *Halophila decipiens* Ostenfeld in estuaries of southwestern Australia. Aquatic Botany 51: 335-340.

Lee SY, Choi CI, Suh Y, Mukai H (2005) Seasonal variation in morphology, growth and reproduction of *Zostera caespitosa* on the southern coast of Korea. Aquat Bot 83: 250-262.

Lipkin, Y., 1979. Quantitative aspects of seagrass communities, particularly of those dominated by *Halophila stipulacea* , in Sinai (Northern Red-Sea). Aquatic Botany, 7(2): 119-128.

Malm, T. (2006). Reproduction and recruitment of the seagrass *Halophila stipulacea* . Aquatic botany, 85(4), 345-349.

Marbà, N., & Walker, D. I. (1999). Growth, flowering, and population dynamics of temperate Western Australian seagrasses. Marine Ecology-Progress Series, 184, 105-118.

McGovern TM (2009) Growth and reproduction of *Ruppia maritima* in the Northern Gulf of Mexico. Gulf of Mexico Science 27: 91-101

McMillan, C., 1981. Seed reserves and seed germination for two seagrasses, *Halodule wrightii* and *Syringodium filiforme* , from the western atlantic. Aquatic Botany, 11: 279-296.

McMillan, C., 1987. Seed-Germination and Seedling Morphology of the Seagrass, *Halophila-Engelmannii* (Hydrocharitaceae). Aquatic Botany, 28(2): 179-188.

Meling-Lopez, A. E., and S. E. Ibarra-Obando. 1999. Annual life cycles of two *Zostera marina* L. populations in the Gulf of California: Contrasts in seasonality and reproductive effort. Aquatic Botany 65:59-69.

Park, J.I. and Lee, K.S., 2009. Peculiar growth dynamics of the surfgrass *Phyllospadix japonicus* on the southeastern coast of Korea. Marine Biology, 156(11): 2221-2233.

Patankar, V., Wagh, T., Tyabji, Z., 2019. Observations on the female flowers and fruiting of tape grass *Enhalus acoroides* from South Andaman Islands, India. Journal of Threatened Taxa, 11(5): 13617-13621.

Peterken CJ, Conacher CA (1997) Seed germination and recolonization of *Zostera capricorni* after grazing by dugongs. Aquat Bot 59: 333-340.

Porter J.L. 2007. Contrasting emergence patterns of *Lamprothamnium macropogon* (Characeae, Charophyceae) and *Ruppia tuberosa* (Potamogetonaceae) from arid-zone saline wetlands in Australia. Charophytes 1:19–27.

Rivers, D. O., Kendrick, G. A., & Walker, D. I. (2011). Microsites play an important role for seedling survival in the seagrass Amphibolis antarctica. Journal of Experimental Marine Biology and Ecology, 401(1-2), 29-35.

Rollon, R. N., Cayabyab, N. M., & Fortes, M. D. (2001). Vegetative dynamics and sexual reproduction of monospecific *Thalassia hemprichii* meadows in the Kalayaan Island Group. Aquatic Botany, 71(3), 239-246.

Shelton, A. O. (2008). Skewed sex ratios, pollen limitation, and reproductive failure in the dioecious seagrass *Phyllospadix* . Ecology, 89(11), 3020-3029.

Short, F.T., Polidoro, B., Livingstone, S.R., Carpenter, K.E., Bandeira, S., Bujang, J.S., Calumpong, H.P., Carruthers, T.J.B., Coles, R.G., Dennison, W.C., Erftemeijer, P.L.A., Fortes, M.D., Freeman, A.S., Jagtap, T.G., Kamal, A.M., Kendrick, G.A., Kenworthy, W.J., La Nafie, Y.A., Nasution, I.M., Orth, R.J., Prathep, A., Sanciangco, J.C., van Tussenbroek, B., Vergara, S.G., Waycott, M. and Zieman, J.C., 2011. Extinction risk assessment of the world's seagrass species. Biological Conservation, 144(7): 1961-1971.

Smith, N. M., & Walker, D. I. (2002). Canopy structure and pollination biology of the seagrasses *Posidonia australis* and *P. sinuosa* (Posidoneaceae). Aquatic Botany, 74(1), 57-70.

Taylor H, Rasheed M 2012. Torres Strait seagrass productivity, climate change resilience and recovery capacity. Proc. 12th Int Coral Reef Symposium, Cairns, Australia.

Thorhaug, A., 1979. Flowering and Fruiting of Restored *Thalassia* Beds - Preliminary Note. Aquatic Botany, 6(2): 189-192.

van Tussenbroek, B.I., Montero, M.M., Wong, R., Santos, M.G.B. and Guzman, J.M., 2010. Pollen limitation in a dioecious seagrass: evidence from a field experiment. Marine Ecology Progress Series, 419: 283-288.

Vollebergh PJ, Congdon RA 1986. Germination and growth of *Ruppia polycarpa* and *Lepilaena cylindrocarpa* in ephemeral saltmarsh pools, Westernport Bay, Victoria. Aquat. Bot. 26: 165-179

Vromans DC, JB Adams, T Riddin (2013) The phenology of *Ruppia cirrhosa* (Petagna) Grande and *Chara* sp. In a small temporarily open/closed estuary. Aquat Bot 110: 1-5.

Watanabe, M., Nakaoka, M. and Mukai, H., 2005. Seasonal variation in vegetative growth and production of the endemic Japanese seagrass *Zostera asiatica* : a comparison with sympatric *Zostera marina* . Botanica Marina, 48(4): 266-273.

Waycott, M., McMahon, K., & Lavery, P. (2014) A guide to southern temperate seagrasses. Csiro Publishing, Australia

Williams, S.L., 1995. Surfgrass (*Phyllospadix torreyi*) reproduction - reprodcutive phenology, resource-allocation, and male rarity. Ecology, 76(6): 1953-1970.

Yabe, t., I Ikusima, T. Tsuchiya. 1995. Production and population ecology of *Phyllospadix iwatensis* Makino. I. Leaf growth and biomass in an intertidal zone. Ecological Research 10:291-299.

Zakaria, M.H., Bujang, J.S. and Arshad, A., 2002. Flowering, fruiting and seedling of annual *Halophila beccarii* Aschers in peninsular Malaysia. Bulletin of Marine Science, 71(3): 1199-1205.

Zhang, X., Zhou, Y., Xu, S., Wang, P., Zhao, P., Yue, S., Gu R., Song X., Xu S., Liu J-X. & Wang, X. (2020). Differences in reproductive effort and sexual recruitment of the seagrass *Zostera japonica* between two geographic populations in northern China. Marine Ecology Progress Series, *638* , 65-81.

SHEET 5

Calculation of the carbon sequestration that would have been delivered by the globally lost seagrass beds

Note that there are uncertainties for each of the numbers used. Also note that the loss of seagrass meadows is accompanied by release of sequestered carbon, which has contributed to the carbon emissions gap today, as well as will contribute to the carbon emission gaps in future; the presented percentage is therefore conservative (e.g. Salinas et al. 2020 online)

Net primary production of seagrass meadow (NPP)	340		g C m-2 y-1	Duarte & Chiscano 1999: 1012 gDW m-2y-1; conversion to Carbon: x 0.336 (Duarte 1990)
15.9% of NPP is buried in seagrass meadow	54		g C m-2 y-1	Duarte & Cebrian 1996
5% of NPP is buried outside seagrass meadow (e.g. deep sea or nearby sediments)	17		g C m-2 y-1	Duarte & Krause-Jensen 2017; Krause-Jensen and Duarte 2016
Imported carbon (from plankton or land-derived)	54		g C m-2 y-1	equal to buried fraction; Kennedy et al. 2010
Carbon sequestration potential of seagrass meadows per m2	125		g C m-2 y-1	sum of previous 3 cells
CO2 sequestration potential of seagrass meadows	459		g CO2 m-2 y-1	conversion molecular weight C -> CO2 factor 3.67
**Range of estimated LOST seagrass area**	**0.25**	**0.30**	**Million km2**	Duarte 2017; Pendleton et al. 2012; respectively
**Not sequestered CO2 because seagrass area was lost**	**0.115**	**0.138**	**Gt CO2 y-1**	

Calculation of the missed contribution of seagrass to the emissions gap 2019-2030 between current policy and the 1.5 and 2.0 oC scenarios (UNEP 2019)

Not sequestered CO2 over the period 2019-2030 (11 year)	1.263	1.515	Gt CO2 y-2	11 x cell B11
**MISSED contribution of lost seagrass area 2019-2030 (1.5 oC scenario 2030)**	**3.61%**	**4.33%**	Gt CO2	35 GtCO2 is the emissions gap in 2030 between current policy and the scenario 1.5 oC (UNEP 2019)
**MISSED contribution of lost seagrass area 2019-2030 (2.0 oC scenario 2030)**	**7.02%**	**8.42%**	Gt CO2	18 GtCO2 is the emissions gap in 2030 between current policy and the scenario 2.0 oC (UNEP 2019)

Literature cited:

Duarte C.M., Cebrian J. 1996. The fate of marine autotrophic production. Limnology and Oceanography 41:1758-1766.

Duarte C.M., 1990. Seagrass nutrient content. Marine Ecology-Progress Series 67:201-207

Duarte C.M., Chiscano C.L. 1999. Seagrass biomass and production: A reassessment. Aquatic Botany 65:159-174.

Duarte, C.M., 2017. Reviews and syntheses: Hidden forests, the role of vegetated coastal habitats in the ocean carbon budget. Biogeosciences, 14(2): 301-310.

Duarte, C.M., Krause-Jensen, D., 2017. Export from seagrss meadows contributes to marine carbon sequestration. Frontiers in Marine Science 4:13

Kennedy, H., Beggins, J., Duarte, C.M., Fourqurean, J.W., Holmer, M., Marbà, N. and Middelburg, J.J., 2010. Seagrass sediments as a global carbon sink: isotopic constraints. Global Biogeochemical Cycles, 24.

Krause-Jensen, D., Duarte, C.M., 2016. Substantial role of macroalge in marine carbon sequestration. Nature Geoscience 9:737-743

Pendleton, L., Donato, D.C., Murray, B.C., Crooks, S., Jenkins, W.A., Sifleet, S., Craft, C., Fourqurean, J.W., Kauffman, J.B., Marbà, N., Megonigal, P., Pidgeon, E., Herr, D., Gordon, D. and Baldera, A., 2012. Estimating global "Blue Carbon" emissions from conversion and degradation of vegetated coastal ecosystems. Plos One, 7(9).

Salinas, C., Duarte, C.M., Lavery, P.S., Masque, P., Arias-Ortiz, A., Leon, J.X., Callaghan, D., Kendrick, G.A. and Serrano, O., Seagrass losses since mid-20th century fuelled CO2 emissions from soil carbon stocks. Global Change Biology.

UNEP, 2019. Emissions Gap Report 2019. Executive summary. United Nations Environment Programme, Nairobi.

SHEET 6

Food production

Edible grains from annual populations of *Zostera marina* provided food to people of Sinaloa, Mexico (Felger & Moser 1973)

seed production	100000	seeds/m2/yr	(Meling-Lopez & Ibarra-Obando 1999)
seed weight	6.5	average mg wet weight per seed	(Felger & McRoy 1975)
**total production**	**6.5**	**ton/ha/yr**	

In comparison: barley gross yields increased from 1 to 2 ton/ha between 1270 and 1870 in Great Britain (Broadberry et al. 2015)

Literature cited:

Felger, R. and Moser, M.B., 1973. Eelgrass (*Zostera marina* L) in Gulf of California - Discovery of Its Nutritional-Value by Seri Indians. Science, 181(4097): 355-356.

Meling-Lopez, A.E. and Ibarra-Obando, S.E., 1999. Annual life cycles of two *Zostera marina* L. populations in the Gulf of California: Contrasts in seasonality and reproductive effort. Aquatic Botany, 65(1-4): 59-69.

Felger, R. and McRoy, C.P. 1975. Seagrasses as potential food plants. In: Somers G.F. (ed.) Seed-bearing Halophytes as Food Plants: Proceedings of a Conference, University of Delaware, DEL-SG-3-75, June 10-11, 1974.

Broadberry S, Campbell BMS, Klein A, Overton M, van Leeuwen B (2015) British Economic Growth 1270-1870. Cambridge University Press
