# Supplementary material for: Rewilding the Sea with Domesticated Seagrass
Source: Bioscience. 2021 Sep 22;71(11):1171–8. doi: 10.1093/biosci/biab092 (PMC8560307; doi:10.1093/biosci/biab092)
Supplement: biab092_Supplemental_Files [file biab092_supplemental_files.zip › Supplementary_Materials.docx]

Rewilding the sea with domesticated seagrass

M.M. van Katwijk, B.I. van Tussenbroek, S.V. Hanssen, A.J. Hendriks, L. Hanssen

Correspondence to: m.vankatwijk@science.ru.nl

Materials and Methods

**Rationale to assess Domestication and Rewilding Potential**

Important traits for *domestication* of seagrass species are (i) large seed production potential that allows for fast reproduction rates, and (ii) harvestability, which implies that seeds can be obtained without damaging the plants, which is assumed to be important when life cycle period is longer than 1 year. Damage is assumed to occur when seeds are located belowground. Potential seed production for a species is derived from extensive literature search and calculated from either maximal reported number of seeds in seedbanks, or from maximal fruit (or ovary) density, and maximal number of ovules per ovary, see supplementary Data S1. Seagrass species have a marked range of potential seed production: between < 10 and > 10,000 seeds m^-2^ y^-1^. Unexpectantly, we did not find a significant (inverse) correlation (r= -0.26, p=0.09, n=43) between maximal potential seed production and seed size. This contrasts with Hendriks & Mulder (2008), who found that for plants and animals in general, the offspring production in numbers is reversely related to the offspring size, yielding a constant total offspring weight m^-2^. In seagrasses, the size of the seeds is strongly related to plant size parameters (e.g. maximum rhizome diameter r=0.50, p<0.0001, n=58), and germination or seedling survival success seem independent of the size of the seeds (Orth et al. 2006). The absence of a correlation between potential seed production and seed size, as well as the absence of a correlation between seed size and germination and seedling survival success, justifies to use potential seed production as the sole assessor of fast reproduction rates.

For *rewilding* it is important that the newly established meadows quickly expand, creating self-sustaining meadows and possibly metapopulations. Thus, important traits for *rewilding* are: i. again seed production potential, ii. life cycle period, which is the period the species can potentially grow from seed and reproduce; this period is ideally short (1 or 2 years) to allow for rapid population growth by seed produced within the rewilded population, and 3. dispersal potential. For the latter, knowledge is only available for selected species (McMahon et al. 2014).

**Method to calculate Domestication and Rewilding Potential (applied in Data S1)**

Domestication potential is the combination of reproductive (i.e. potential seed production) and harvest potential (yes or no).

Calculation: Domestication potential = Potential seed production or zero (if seeds are released belowground *and* life cycle period more than one year).

Rewilding will be potentially exponential, thus the inverse of the life cycle period is in the exponent.

Calculation: Rewilding potential = (Potential seed production)^1/life cycle period^

Both values were log-transformed for visibility in the plot. Grouping: We tentatively grouped the seagrass species in high, intermediate and low potential for domestication and rewilding, numerical criteria see Data S1 sheet 1). In addition we distinguish a group with high or intermediate domestication potential (high potential seed production), but low rewilding potential due to a long life cycle period.

Note that (1) species with lower seed production potential could still be suitable for rewilding through vegetative spread (e.g. Smulders et al. 2017), this is not taken into account in the present study, and (2) that the assessment of potential for domestication and rewilding may change upon further research, particularly since it is based on maximum values; maxima are inherently influenced by the number of observations.

**References cited**

1. Hendriks, A.J. and Mulder, C., 2008. Scaling of offspring number and mass to plant and animal size: model and meta-analysis. Oecologia, 155(4): 705-716.
2. McMahon, K., van Dijk, K.J., Ruiz-Montoya, L., Kendrick, G.A., Krauss, S.L., Waycott, M., Verduin, J., Lowe, R., Statton, J., Brown, E. and Duarte, C., 2014. The movement ecology of seagrasses. Proceedings of the Royal Society B-Biological Sciences, 281(1795).
3. Orth, R.J., Harwell, M.C. and Inglis, G.J., 2006. Ecology of seagrass seeds and seagrass dispersal processes. In: A.W.D. Larkum, R.J. Orth and C.M. Duarte (Editors). Springer, Dordrecht, The Netherlands, pp. 111-133.
4. Smulders, F.O.H., Vonk, J.A., Engel, M.S. and Christianen, M.J.A., 2017. Expansion and fragment settlement of the non-native seagrass Halophila stipulacea in a Caribbean bay. Marine Biology Research, 13(9): 967-974.
